# Effect of liquid nitrogen pre-treatment on various types of wool waste fibres for biogas production

**DOI:** 10.1016/j.heliyon.2018.e00619

**Published:** 2018-05-02

**Authors:** Elena Kuzmanova, Nikolai Zhelev, Joseph C. Akunna

**Affiliations:** a School of Science, Engineering and Technology, Abertay University, Bell Street, Dundee DD1 1HG, UK; b Urban Water Technology Centre, School of Science, Engineering and Technology, Abertay University, Bell Street, Dundee DD1 1HG, UK

**Keywords:** Environmental science, Energy, Materials science

## Abstract

This study investigated the role of liquid nitrogen (LN_2_) in increasing microbial accessibility of wool proteins for biogas production. It involves a mechanical size reduction of four different types of raw wool fibres, namely, Blackface, Bluefaced Leicester, Texel and Scotch Mule, in presence of liquid nitrogen, followed by the determination of the methane production potential of the pre-treated wool fibres. The highest methane yield, 157.3 cm^3^ g^−1^ VS, was obtained from pre-treated Scotch mule wool fibre culture, and represented more than 80% increase when compared to the yield obtained from its raw equivalent culture. The increase in biogas yield was attributed to the effectiveness of LN_2_ in enhancing particle size reduction and the consequent increase in wool solubility and bioavailability. Results also showed that LN_2_ pre-treatment can enhance size reduction but has limited effect on the molecular structure. The study also showed that the biogas potential of waste wool fibres varies with the type and source of wool.

## 1 Introduction

Wool is natural fibre material obtained majorly by shearing sheep and generally used in the textile manufacturing. The world textile industry has largely amplified the production due to the increasing global need for manmade fibres estimated from 52.6 million tonnes in year 2000 to 70.5 million tonnes in 2009 and surpassed the volume of 100 million tonnes in 2016, where wool is 1.56% from the total world fibre production [1, 2, 3]. The sheep population in the European Union (EU) area is constantly increasing and now is the second largest, amounted to about 90.4 million, with the majority of the sheep located in the United Kingdom (UK) [4].

Wool waste is also available on international markets in abundant quantities. In the UK in 2009 only the clothing industry generated 164,984 tonnes of wool waste, sourced from fibre production, processing, garment production, distribution and import, retail, use and end-of-life [5].

In addition, disposal of wool wastes to landfill is banned due to the potential environmental pollution and risk associated with the spread of contagious diseases. Various industries are interested of wool waste, due to its properties, for development of thermal and sound insulation materials [6]. Other efforts were dedicated to value-added utilisation of wool waste or hydrolysate as a fertiliser [7, 8] or as a chelating agents [9]. However, there is an increasing interest in treating wool for energy production. Anaerobic digestion offers a sustainable technology for treating wool waste, which has a great potential to both reduction of waste and energy generation.

Wool is primarily constituted of a broad category of insoluble proteins, referred to as “keratins”. Keratins are fibrous proteins containing amino acid groups bond into crystalline structure and displaying high stability and low solubility. This type of bonding provides resistance to environmental, chemical and enzymatic degradation, and poses a challenge for anaerobic biodegradation processes. Its structure also contains a large amount of sulphur containing amino acid cysteine, which further limits its biodegradability. Consequently, only limited investigation on the anaerobic digestibility of wool has been reported [1, 10, 11], and reported studies have been mainly on chemical or enzymatic pre-treatment. Combined thermal and enzymatic pre-treatment of wool materials and fabrics followed by thermophilic anaerobic digestion have been reported to deliver up to 20 times higher methane yield than untreated samples [1], whilst thermal treatment alone presented a considerable lower methane yields [10].

The effect of mechanical pre-treatment of wool for mesophilic biogas production has not been sufficiently addressed in the literature. This may be because wool cell destruction has been identified as being capable of delaying or obstructing the process of biodegradation due to the complex bond of protein and fibre in the wool structure [12]. This study attempts to overcome this obstacle by employing cryogen-cooling methods of liquid nitrogen to aid mechanical pre-treatment. LN_2_ has been successfully used for cell wall destruction of microalgae to increase the release of lipids for biodiesel production [13]. The aim of this study therefore is to explore the effect of LN_2_ pre-treatment of various types of raw wool waste in a mesophilic anaerobic digestion process. Various qualitative and quantitative methods including electrophoresis were also employed in the study to determine the effects of the LN_2_ treatment on the wool chemical structure.

## 2 Materials and methods

**Fig. 1:**
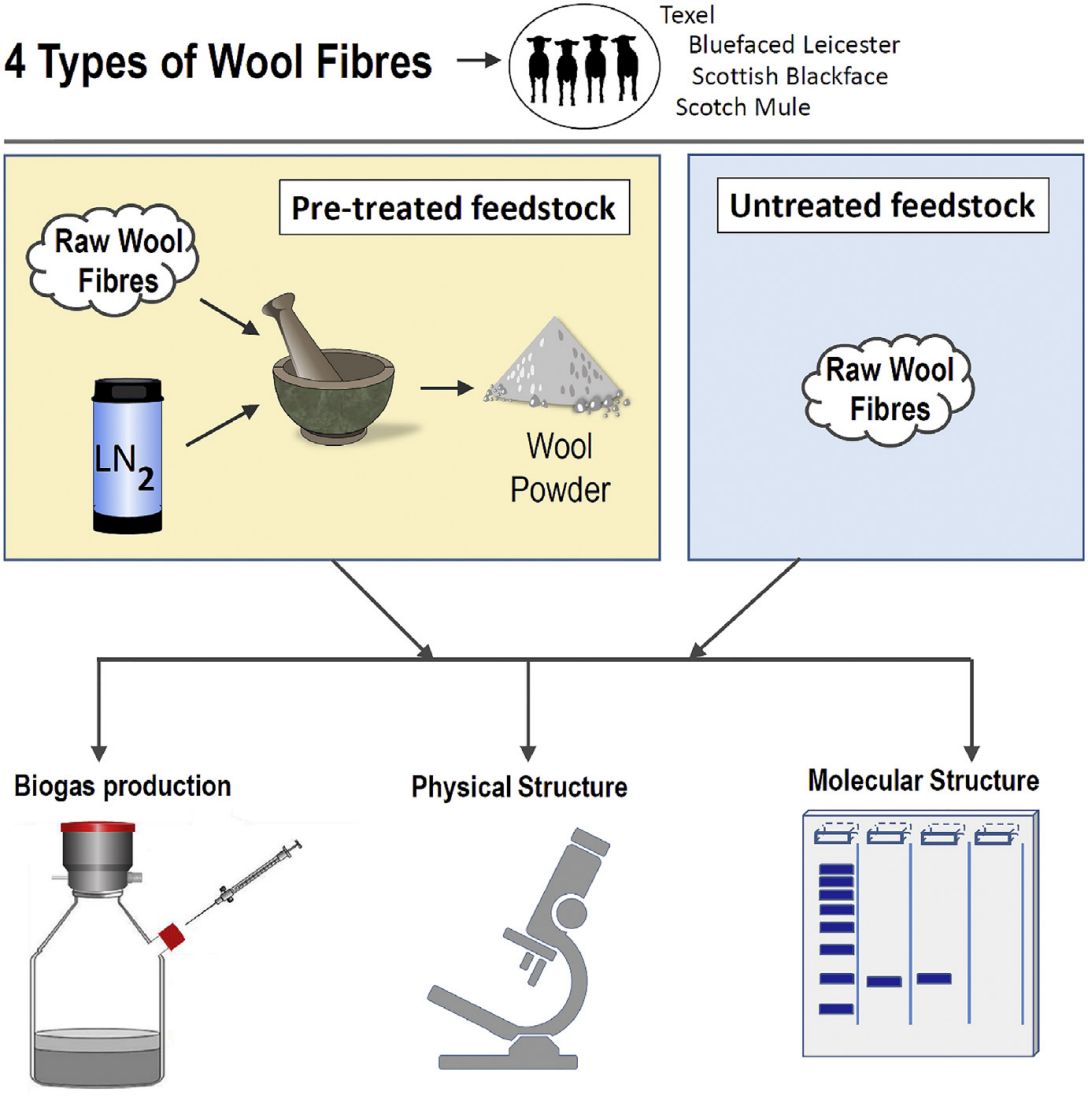
Methodology employed in the study.

The methodology employed in this study is presented in Fig. 1.

### 2.1 LN_2_ pre-treatment

**Fig. 2:**
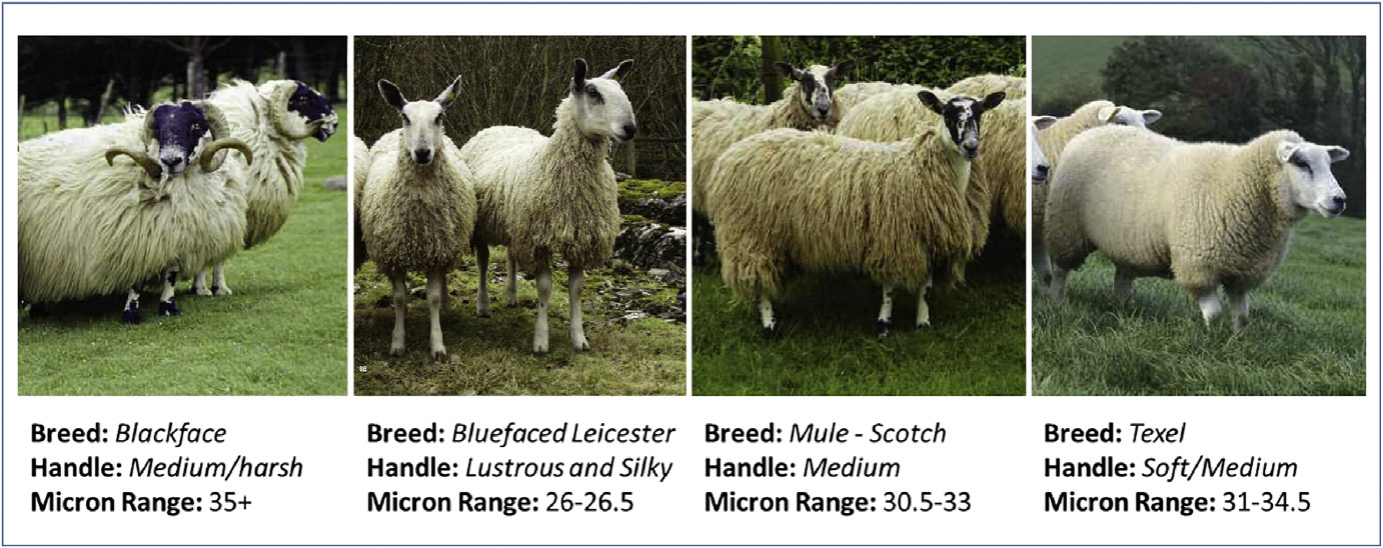
Wool types used for the experiment (BWMB 2016**).**

**Fig. 3:**
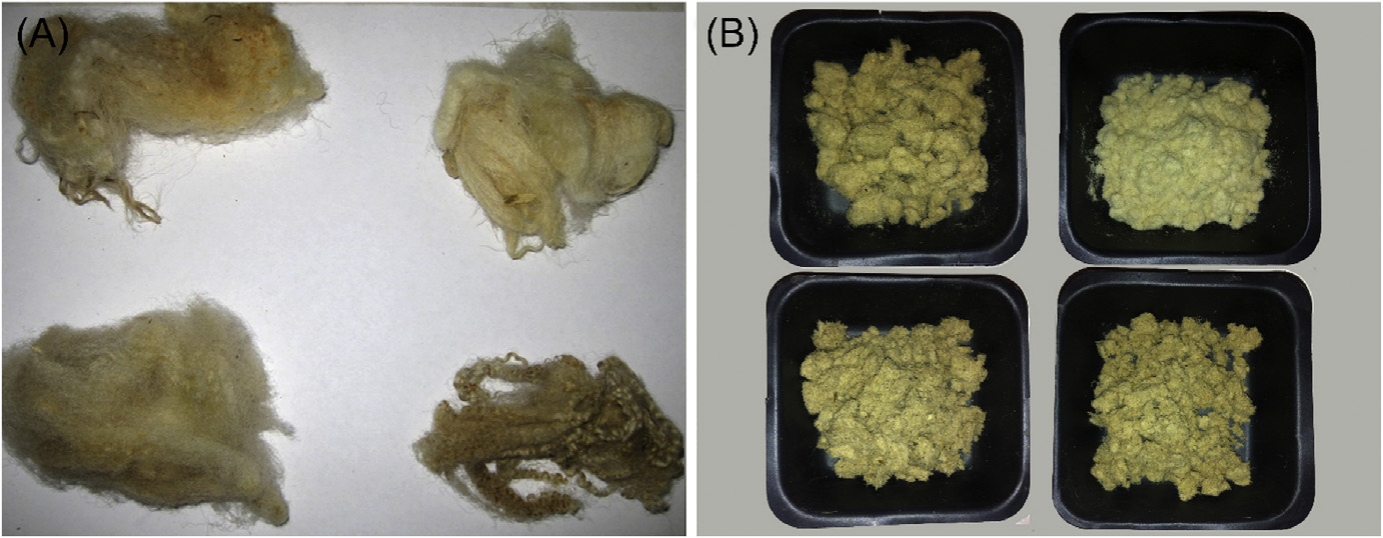
Top left – Scotch Mule, top right – Scottish Blackface, bottom left – Texel, bottom right – Blue Faced Leicester. (A) Raw wool samples (B) After LN_2_ treatment.

Wool fibres were obtained after sheep shearing from various sheep (*Ovis aries*) breeds in the summer period of 2014 from a farm in Angus area, in North East Scotland (56.7 N, −3.17 W). Four different types of wool fibres, namely, Blackface, Bluefaced Leicester, Texel and Scotch Mule, were utilised in the experiment, with variation in the type and size of the fibres, as shown in Fig. 2 [14]. The raw wool fibres were stored into sealed plastic bags at ambient temperature while transferred to the lab and until utilisation. A portion of each of the samples were manually grinded in a mortar and pestle with application of LN_2_, until reaching homogenised powder form. The quantity of liquid nitrogen for the production of 20 g wool powder varied widely depending on the type of wool, with the lowest quantity of about 3 l for the Scottish Blackface (hand cleaned) wool fibre. The physical appearances of the samples, with and without pre-treatment with LN_2_ are shown in Fig. 3. The produced organic material was stored in plastic bags and used in a powdered form when required.

### 2.2 Biochemical methane potential (BMP) test

The experimental design employed for the BMP test was based on a method developed by [15]. The experiments were carried out in 0.5 l capacity bottles in Ankom^RF^ gas production system (Ankom^RF^ Technology, USA). Culture bottles were each added 0.4 l of settled seed inoculum and 5 g wool powder. The experiments were carried out using pre-treated and raw wool fibres. Seed inoculum was obtained from a mesophilic anaerobic digester (Hatton, Angus, Scotland) treating municipal wastewater sludge. The controls or blanks culture bottles containing only 0.4 l inoculum were also prepared. The bottles were stored in incubator at 37 °C for a period of 40 days, with intermittent shaking.

### 2.3 Method of analysis

#### 2.3.1 Methane gas

Ankom^RF^ digestion system measures the cumulative biogas pressure production. The methane content was determined (daily up to Day 10 and thereafter routinely), using a gas chromatograph (Hewlett Packard (HP) 5890 Series II). Gas samples were collected in gas tight syringes and 100 mm^3^ of each sample analysed using a Gas Chromatograph (GC). Overpressure was calculated by the Ankom^RF^ system and volume recorded every hour. Overall results take into account the cumulative pressure inside the bottles, overpressure released and methane percentage. The amount of methane gas produced was calculated under Standard Temperature and Pressure (STP). The pH levels of the culture media were determined periodically using a HACH-SensION 3.

#### 2.3.2 Protein separation and characterisation

##### 2.3.2.1 Extraction of wool protein

The wool fibre fragments of the raw untreated samples were cut with scissors, whereas the LN_2_ pre-treated samples were already in a powdered form. Each sample was washed with ethanol and then with a mixture of chloroform/methanol (2:1, v/v) for 24 hours to remove external lipids. The modified Shindai method [16] was used for the extraction of protein. 20 g delipidised wool fibres was mixed with solution (5 cm^3^) containing 3.94 kg m^−3^ Tris–HCl, pH 8.5, 197.9 kg m^−3^ thiourea and 300.3 kg m^−3^ urea. After incubation for 3 days at 50 °C, the mixture was centrifuged at 15,000 rpm for 30 min at 20 °C in Millipore UF4BG00 tubes (Eppendorf Centrifuge 5804R). The supernatant was recovered and used for quantification of the protein concentration and for the electrophoresis experiment.

##### 2.3.2.2 Quantification of the extracted wool protein

Bradford protein assay method (BioRad) was used for the determination of dissolved wool proteins, using bovine serum albumin as standard.

##### 2.3.2.3 Gel electrophoresis

Initially, 200 mm^3^ sample buffer was added into each Eppendorf tube, which contained the mixture from the extraction procedure and boiled using the Thermomixer compact for 15 minutes. The sample was then sonicated at 50% amplitude (QSonica, Q55, USA) for 15 seconds each in order to facilitate protein solubilisation and then washed with distilled water. Sodium Dodecyl Dulfate-Polyacrylamide Gel Electrophoresis SDS-PAGE was performed according to the Laemmli's method [17] using XcellSureLock^TM^ Mini-Cell (Invitrogen), on Bolt^TM^ 4-12% BT plus 10w, 12% polyacrilamide gels. Proteins in gel were stained with 0.1% Coomassie brilliant blue R-250, 10% acetic acid and 40% methanol for 3 hours and de-stained in 10% acetic acid and 40% methanol until the bands were clearly noticeable.

##### 2.3.2.4 Microscopy

The physical wool fibre disruption was examined using inverted light microscope (Leica HC) under different magnification levels.

## 3 Results and discussion

### 3.1 Effect of LN_2_ treatment on the molecular structure

**Fig. 4:**
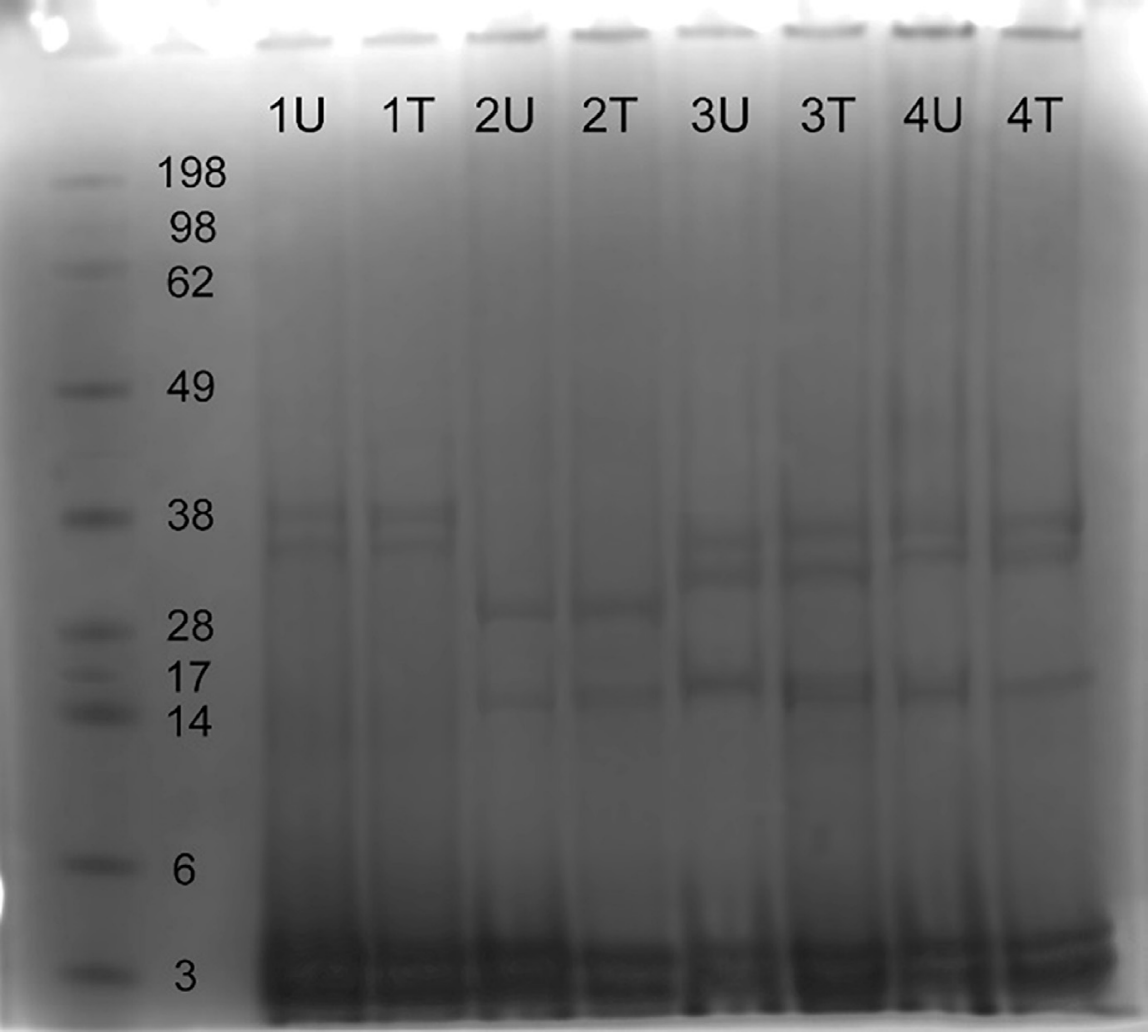
The effect of urea concentration on the solubilisation of the proteins from wool fibres: *U* corresponds to raw, untreated sample, where *T* is LN_2_ treated sample. (1) Blackface (hand cleaned) (2) Scotch Mule (3) Texel (4) Bluefaced Leicester.

Urea-based extraction procedure was applied in order to avoid masking the changes from LN_2_ treatment, and the result is shown in Fig. 4. The electrophoretical analysis pattern of wool proteins for Blackface, Bluefaced Leicester and Texel sheep breeds showed similar pattern producing two high molecular mass bands. The degraded proteins consisted of microfibrillar keratins with a molecular mass of 38 kDa. Keratin-associated proteins (KAPs) of 14–28 kDa presented visible bands in Bluefaced Leicester, Scotch Mule and Texel, whereas in Blackface were absent. Two distinctive low molecular mass bands of glycerine-tyrosine rich proteins with molecular weight of 3–6 kDa, were present in each wool fibre from all sheep breeds from results obtained from sodium dodecyl sulfate-polyacrylamide gel electrophoresis (SDS-PAGE). In sole denaturant, urea based protein buffer, the extraction of the keratins, glycerine-tyrosine rich proteins and KAPs was prompted, while the keratins were suppressed. This is the result of the robust structure of keratins and gentle environmentally friendly pre-treatment urea associated degradation, instead of detergent or mercaptoethanol application. In general, the molecular structure of keratins remained intact and unchanged after LN_2_ treatment. There was however, evidence of greater level of homogenising effect of the treatment.

### 3.2 Effect of LN_2_ pre-treatment on the wool morphology and solubility of protein

**Fig. 5:**
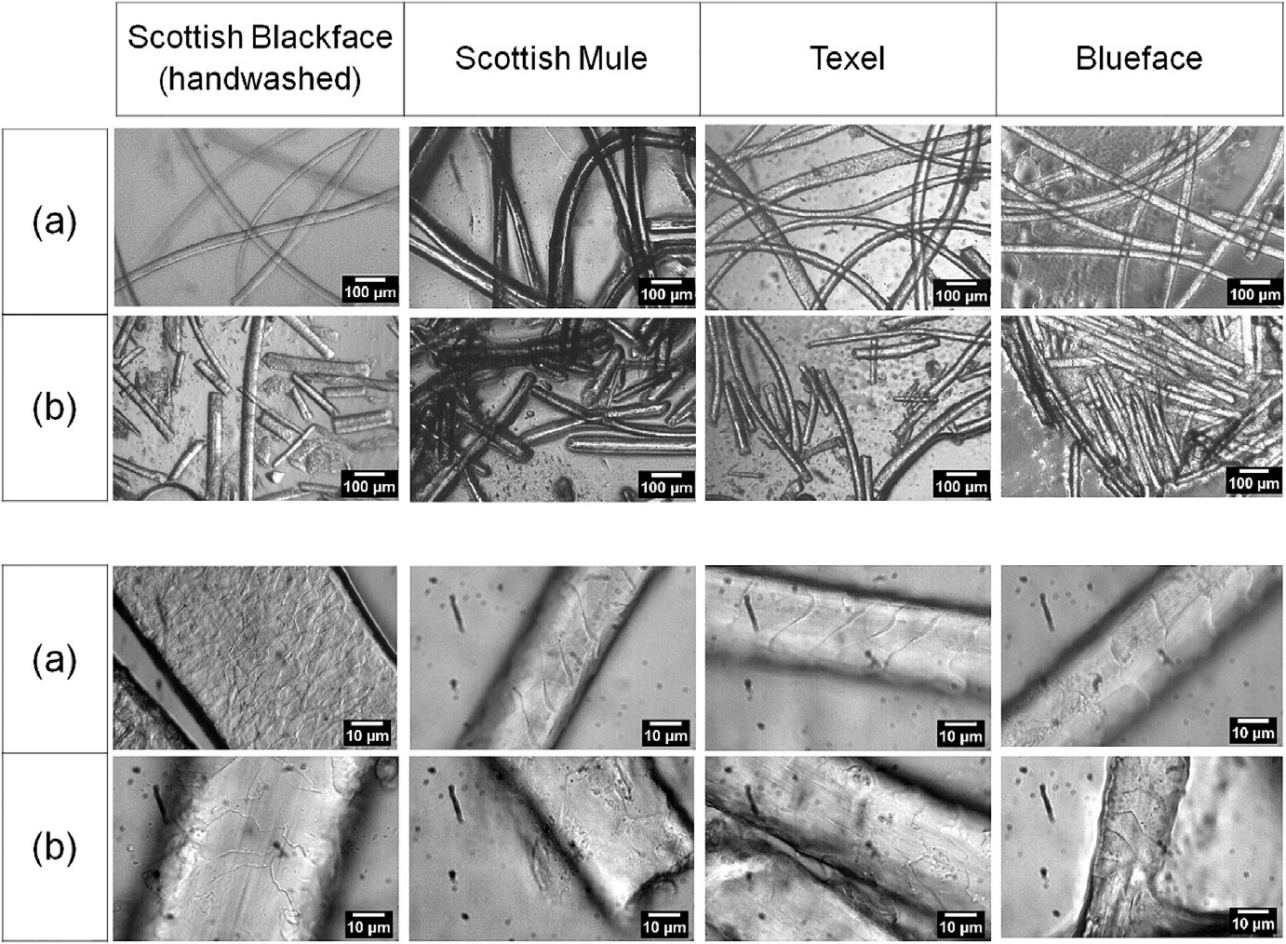
Morphology of the wool samples (a) before and (b) after LN_2_ treatment.

LN_2_ treatment appeared to breakdown the wool fibre randomly as shown in Fig. 5. The surface of the wool samples after treatment retained the macrostructure with visible changes in outer morphology of the fibres. Physical structural changes of the wool fibre morphology by unscaling and peeling off effect on the outer surface of the wool fibre were observed. The scales of the cuticle cells of the fibre appeared smoothened and even compared to the untreated raw wool. There was also evidence of size reduction to sizes in the range of approximately 100 μm–400 μm. In general, it appears that the treatment resulted in an increase in surface area, which can enhance water permeability and weakening the structural bonds within the fibres, thereby facilitating resulting to increased solubility and microbial activity. Increase in surface area can also lead to more surface being accessible to microbial action, and hence, will result in higher rates of biodegradation.

LN_2_ treatment also brought about significant increases in solubility of protein amounting to 14%, 38%, 32% and 37% respectively for Scottish Blackface-hand cleaned, Scotch Mule, Texel and Bluefaced Leicester. The real implication of the LN_2_ pre-treatment applied on various wool fibres is that the molecular structure remains unchanged, whereas alterations by physical process of scaling off the outer surface and considerable size reduction, contributed to the larger surface area exposure, facilitating organic material break down by bacteria. These physical structural changes affect samples solubility, allowing the proteins to be extracted and utilized in the AD process.

### 3.3 Effect of LN_2_ pre-treatment on methane production

**Fig. 6:**
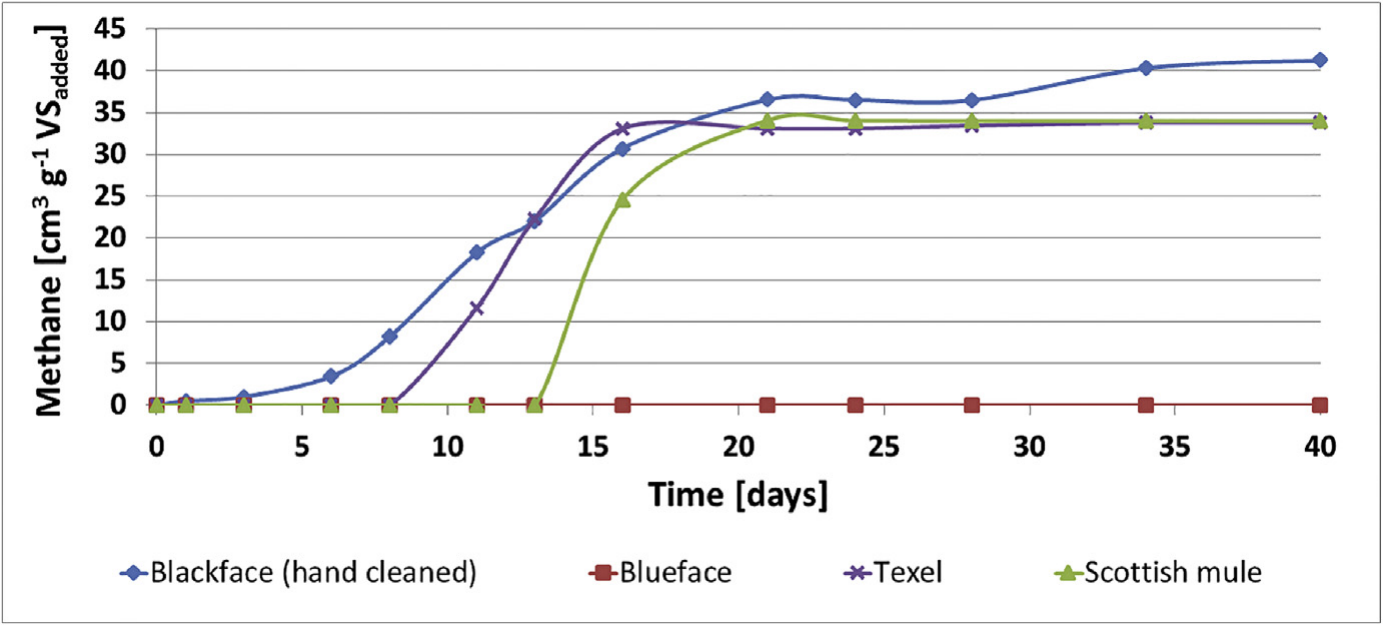
Cumulative methane production by raw wool cultures.

For raw wool fibres, methane production varied significantly during start-up and all through the digestion period, as shown in Fig. 6. Blackface handcleaned wool culture produced the highest methane of 41.3 cm^3^ g^−1^ VS whilst Bluefaced Leicester culture did not produce detectable levels of methane during the experimental period. The Scotch mule and Texel cultures experienced delayed start-up and produced similar amounts of methane of 34.8 and 33.8 cm^3^ g^−1^ VS, respectively. The pH value of all cultures was about 7.5, and the VFA values were in the range of 83–102 mg L^−1^ of (HOACs) at the end of the experimental period. The lowest VFA concentration was observed in the Bluefaced Leicester wool culture.

**Fig. 7:**
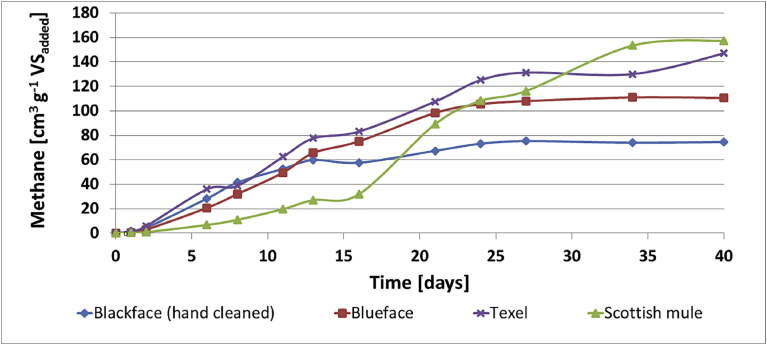
Cumulative methane production by LN_2_ pre-treated wool cultures.

The methane production of LN_2_ treated wool cultures are shown in Fig. 7. In comparison with Fig. 6, all LN_2_ pre-treated wool cultures experienced faster start-up and produced higher amount of methane gas. Furthermore, unlike in Fig. 6, methane gas was produced in the Bluefaced Leicester wool culture. The maximum methane production of 157.3 cm^3^ g^−1^ VS, was obtained in Scotch mule culture followed by Texel, Bluefaced Leicester and Scottish Blackface (hand-cleaned), producing 147.0, 110.6, 74.7 cm^3^ g^−1^ VS, respectively. The pH value of all cultures was in the range of 7.4–7.5 and the VFA values in the range of 90–160 mg L^−1^ of (HOACs) at the end of the experimental period.

**Fig. 8:**
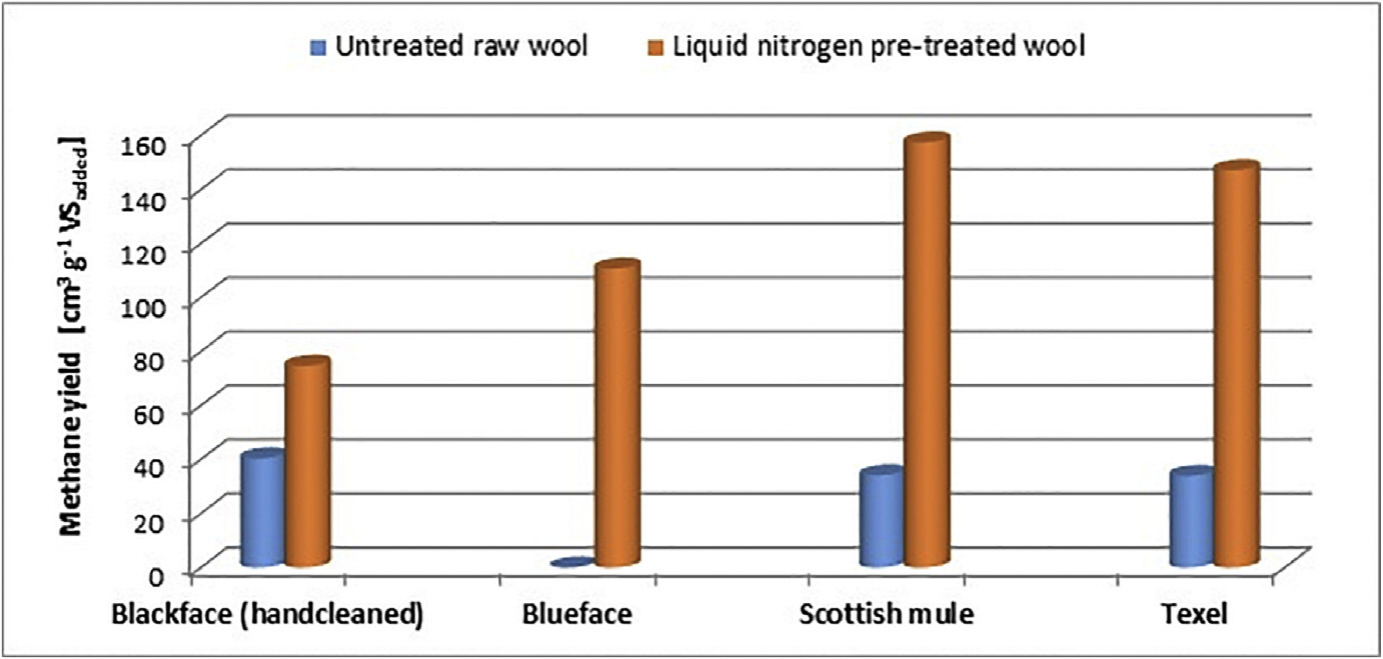
Methane yields of various treated and untreated wool fibres.

Figs. 6 and 7 show that methane production potential of raw wool is depended on the type of wool and is within the range of 0–40 cm^3^ g^−1^ VS, The result is consistent with estimates reported in the literature for untreated keratinous waste [1, 10]. This study has also shown that pre-treatment with LN_2_ can increase the methane production potential to 75–157 cm^3^ g^−1^ VS. For the four types of wool used in this study, LN_2_ pre-treatment led to faster start-up and to 46%, 100%, 78% and 77% increases in methane yield for the Blackface (hand-cleaned), Bluefaced Leicester, Scotch Mule and Texel wool fibres. All the LN_2_ pre-treated wool fibres produced higher amount of methane gas as shown in Fig. 8.

## 4 Conclusion

This study has shown that pre-treatment of wool fibres can enhance the amenability of wool fibres to anaerobic biodegradation, thereby contributing to increased biogas yield. Results also suggest that LN_2_ pre-treatment can lead to changes in the physical structure of the fibres, and may not bring about significant alterations in the molecular structure. The pre-treatment brings about greater particle size reduction, and in so doing enhances their solubility and bioavailability for microbial degradation. In this study, LN_2_ pre-treatment has been found effective in improving the anaerobic biodegradability of wool waste fibres, resulting in up to 80% increase in biogas production. The study also revealed that the biogas potential of wool waste fibres is dependent on the type and source of wool.

## Declarations

### Author contribution statement

Elena Kuzmanova: Performed the experiments; Analyzed and interpreted the data; Wrote the paper.

Nikolai Zhelev: Conceived and designed the experiments; Analyzed and interpreted the data; Contributed reagents, materials, analysis tools or data.

Joseph Akunna: Conceived and designed the experiments; Analyzed and interpreted the data; Contributed reagents, materials, analysis tools or data; Wrote the paper.

### Funding statement

This work was supported by Abertay University, Dundee, UK.

### Competing interest statement

The authors declare no conflict of interest.

### Additional information

No additional information is available for this paper.
